# Testing the Paradox of Enrichment along a Land Use Gradient in a Multitrophic Aboveground and Belowground Community

**DOI:** 10.1371/journal.pone.0049034

**Published:** 2012-11-08

**Authors:** Katrin M. Meyer, Matthijs Vos, Wolf M. Mooij, W. H. Gera Hol, Aad J. Termorshuizen, Wim H. van der Putten

**Affiliations:** 1 Department of Terrestrial Ecology, Netherlands Institute of Ecology NIOO-KNAW, Wageningen, The Netherlands; 2 Ecosystem Modelling, Faculty of Forest Sciences and Forest Ecology, University of Goettingen, Göttingen, Germany; 3 Department of Ecology & Ecosystem Modeling, Institute of Biochemistry and Biology, University of Potsdam, Potsdam, Germany; 4 Department of Aquatic Ecology, Netherlands Institute of Ecology NIOO-KNAW, Wageningen, The Netherlands; 5 Aquatic Ecology and Water Quality Management, Department of Environmental Sciences Group, Wageningen University, Wageningen, The Netherlands; 6 BLGG Research, Wageningen, The Netherlands; 7 Laboratory of Nematology, Wageningen University, Wageningen, The Netherlands; Centro de Investigación y de Estudios Avanzados, Mexico

## Abstract

In the light of ongoing land use changes, it is important to understand how multitrophic communities perform at different land use intensities. The paradox of enrichment predicts that fertilization leads to destabilization and extinction of predator-prey systems. We tested this prediction for a land use intensity gradient from natural to highly fertilized agricultural ecosystems. We included multiple aboveground and belowground trophic levels and land use-dependent searching efficiencies of insects. To overcome logistic constraints of field experiments, we used a successfully validated simulation model to investigate plant responses to removal of herbivores and their enemies. Consistent with our predictions, instability measured by herbivore-induced plant mortality increased with increasing land use intensity. Simultaneously, the balance between herbivores and natural enemies turned increasingly towards herbivore dominance and natural enemy failure. Under natural conditions, there were more frequently significant effects of belowground herbivores and their natural enemies on plant performance, whereas there were more aboveground effects in agroecosystems. This result was partly due to the “boom-bust” behavior of the shoot herbivore population. Plant responses to herbivore or natural enemy removal were much more abrupt than the imposed smooth land use intensity gradient. This may be due to the presence of multiple trophic levels aboveground and belowground. Our model suggests that destabilization and extinction are more likely to occur in agroecosystems than in natural communities, but the shape of the relationship is nonlinear under the influence of multiple trophic interactions.

## Introduction

Land use change currently is the most important cause of terrestrial biodiversity loss [1]. One of the most outspoken changes is that nutrient-poor natural ecosystems are transformed into highly fertilized agroecosystems. Thus, predicting plant community responses to land use change requires understanding how plant species [2] and their associated aboveground and belowground trophic networks [3] respond to changes in resource availability and vegetation structure. The paradox of enrichment predicts that fertilization leads to a destabilization of population dynamics. The ensuing boom-bust cycles may lead to the extinction of predators and/or their prey [4]. Testing the paradox of enrichment in a complex community with multiple trophic levels requires species exclusion experiments that are difficult to implement in the field or greenhouse, but are highly amenable to modeling approaches [5]. In line with claims for more integration between models and field studies in aboveground-belowground interactions research [3], [6], we use a successfully validated simulation model [7] to test the paradox of enrichment along a land use intensity gradient including multiple trophic levels aboveground and belowground.

Predator-prey theory predicts that stable coexistence of predators and preys is possible if their population dynamics are not too closely coupled [8]. The stability of a predator-prey system can depend on factors such as searching efficiency of the predator or resource availability to the prey. According to the paradox of enrichment, high resource availability leads to an explosion of prey populations causing a sudden increase in predator populations which may first drive the prey and subsequently the predators to extinction. It is also possible that the predator goes locally extinct following the major population decline of its prey, after which the prey population explodes back up, now unchecked by predator-induced mortality. Stochastic processes determine whether only the predator or both predator and prey go extinct. This implies that enrichment increases uncertainty in how the relative performance of herbivores and natural enemies will turn out to affect the fate of plant populations. During land use change, fertilization often goes along with increased insect searching efficiencies, because searching for food plants and host species is facilitated in structurally poor agricultural monocultures (e.g. [9]–[11]). The Rosenzweig-MacArthur model predicts that high predator searching efficiencies will destabilize predator-prey systems [12], because high predator efficiency increases the coupling between predator and prey population dynamics. In the simple bi-trophic Rosenzweig-MacArthur model, this is the case when the predator zero-growth isocline is located at very low prey densities. Also in more complex food chain models [13], predator efficiency is an important factor affecting stability. We may therefore expect enhanced destabilization in agricultural systems where both fertilization and searching efficiencies are high. We also expect that destabilization will increase smoothly, in a non-abrupt way, if land use change is smooth and linear.

Predator-prey theory has been tested for two trophic levels, and occasionally for tritrophic systems [13]–[14]. However, plants are involved in both aboveground and belowground multitrophic level interactions [15] and much is unknown about the applicability of predator-prey theory to multiple trophic levels aboveground and belowground. Results obtained in a simple predator-prey system or food chain will not necessarily hold for more complex communities and food webs. In a trophic network with plants at the first trophic level, herbivores at the second, parasitoids at the third and hyperparasitoids at the fourth level, the natural enemies at the third trophic level can enhance agricultural production as biological control agents. The third trophic level is also central in the debate of top-down versus bottom-up control in natural food webs [16]. The efficiency of biological control depends on the relative susceptibility of herbivores and natural enemies to land use changes.

Disentangling the effects of aboveground and belowground control [17]–[18] could help to design more efficient and better targeted biological control schemes. Most studies comparing aboveground with belowground interactions showed dominant aboveground effects and weak belowground effects on plants [19]–[24]. On the other hand, a few studies indicated strongest trophic effects belowground [7], [25]–[26]. Predator-prey theory predicts that the impact of aboveground and belowground natural enemies of herbivores is strongly related to the tendency of these populations to cycle. Population fluctuations may be larger aboveground, because air is more permeable to movement than soil, so that insect searching efficiencies can be much higher. This may result in larger increases and declines in population density. This effect would be enhanced by fertilization, so that we expect an increased variability in aboveground versus belowground interactions with increasing land use intensity. This increased variability may increase the maximum effect that aboveground interactions can have on plant performance.

The overall aim of the present study was to investigate whether the predictions of the paradox of enrichment hold when multiple aboveground and belowground trophic levels and their searching efficiencies are included, and how the results relate to plant performance. We used herbivore-induced plant mortality as a proxy for system instability and assessed it along a land use intensity gradient from low to high fertilization levels and insect searching efficiencies. We derived three predictions from predator-prey theory and tested them using an individual-based simulation model of aboveground-belowground multitrophic interactions [7]: (1) Fertilization and increasing searching efficiencies lead to destabilization of the system, i.e. to increased plant mortalities, (2) destabilization is stronger for aboveground than for belowground interactions, and (3) destabilization is changing smoothly, i.e. non-abruptly, with land use intensity. We found support for the first two predictions that destabilization increases with land use intensity and that it is stronger aboveground than belowground. However, contradicting the third prediction there were abrupt changes in destabilization with land use intensity.

## Materials and Methods

### The Model

The individual- and rule-based ABove-BElowground interactions model ABBE was developed to investigate plant performance in a food web of plant shoots, herbivores, parasitoids and hyperparasitoids aboveground and plant roots, herbivores and their antagonists belowground [7]. The model also included earthworms that have positive effects on plants without being part of the plant-based food web. Aboveground interactions with the plant are modeled on an individual-by-individual basis, while belowground interactions are considered at the level of populations [7]. One single plant individual is modelled at the centre of aboveground and belowground interactions in each simulation to allow comparisons with the experimental conditions in Soler et al. [27], which provided most of the parameter values for ABBE.

The plant is simulated according to the wild cruciferous plant *Brassica nigra* L. (Brassicaceae), the aboveground herbivores are represented by the specialist chewing larvae of *Pieris brassicae* L. (Lepidoptera: Pieridae) and the belowground herbivores are represented by the specialized root-feeding larvae of the cabbage root fly *Delia radicum* L. (Diptera: Anthomyiidae). Aboveground, the third and fourth trophic levels are parameterized according to the gregarious koinobiont endoparasitoid *Cotesia glomerata* L. (Hymenoptera: Braconidae) and its secondary hyperparasitoid, the solitary idiobiont *Lysibia nana* Gravenhorst (Hymenoptera: Ichneumonidae). As belowground third trophic level, we added the coleopteran egg predator *Aleochara billineata* Gyll. (Coleoptera: Staphylinidae) to the system of Soler et al. [27]. The earthworm component of the community was parameterized with data from the species *Aporrectodea caliginosa* Savigny (Lumbricidae) and *Octolasion tyrtaeum* Savigny (Lumbricidae) (see [7]).

The timeframe of the simulations is one growing season, the general time step is six weeks, space is not considered explicitly. The simulated processes in any one time step are nutrient pool replenishment, plant growth, earthworm action, shoot herbivore mortality, parasitoid mortality and reproduction, shoot biomass reduction by herbivores, parasitism of herbivores by parasitoids, hyperparasitoid mortality and reproduction, parasitism of parasitoids by hyperparasitoids, plant mortality due to shoot herbivory, belowground antagonist mortality and reproduction, root herbivore mortality due to antagonist attack, root herbivore natural mortality, root biomass reduction by root herbivores, plant mortality due to root herbivory, shoot and root herbivore reproduction [7]. We assume that the only cause of plant mortality is excessive herbivory and that shoot herbivore efficiency depends on parasitism, whereas parasitoid efficiency is independent of parasitism by hyperparasitoids [7]. Shoot herbivore efficiency is the proportional shoot biomass decrease per individual and parasitoid efficiency is parasitizing success probability (Table 1). Shoot herbivore presence increases shoot quality [27] and induces shoot volatile emission, increasing parasitism efficiency. Shoot quality is standardized to range from 0 to 1 and shoot volatile emission can only be turned on or off in the model. Individual herbivore body size depends on shoot quality while body sizes of higher trophic levels depend on the body size of their hosts. Egg load of aboveground trophic levels depends on body size. ABBE has successfully been validated [7] against independent experimental data (e.g. [27]). Further details on structure and parameterization of ABBE can be obtained from [5],[7].

**Table 1:** Composition of the land use intensity gradient from low to high fertilization and searching efficiencies.^a^

Trophic level	Property	Model parameter [unit]	Parameter value	
			Low	High
Abiotic	Fertilization	Nutrient supply [proportion of 100% nutrient supply]	0	1
AG parasitoid	Efficiency	Parasitizing success probability [probability]	0.092	0.788
BG herbivore	Efficiency	Proportional root biomass decrease per individual [proportion]	0.0002	0.0667
BG antagonist	Efficiency	Maximum number of BG herbivore eggs killed per antagonist couple lifetime [numbers]	260	1438
AG herbivore	Load	Initial number of AG herbivores [numbers]	0	2580
AG herbivore	Mortality	AG herbivore natural mortality [probability]	0.9975	0.785
Plant	Quality increase due to herbivory	Shoot quality increase due to defense induction by AG herbivores [proportion of 100% quality]	0.93	0.0275

a The parameters plant quality and aboveground herbivore mortality are linearly decreasing with increasing land use intensity, the others are linearly increasing. All parameters were changed at the same time. AG – aboveground, BG – belowground.

### Land Use Intensity Gradient

Plant mortality and biomass was investigated along a simulated increase in land use intensity consisting of fertilization and searching efficiency parameters (Table 1). These parameters represented a gradient from structurally complex, unfertilized natural systems to fertilized agricultural monocultures (see e.g. [9]–[11]). Greater searching efficiencies of insects were simulated by increasing insect numbers and efficiencies at all trophic levels, decreasing herbivore natural mortalities, and reducing the positive impact of herbivory on shoot quality (Table 1; the positive impact of herbivory on shoot quality was originally reported by [27] for nutrient-poor conditions). We included parameters of all trophic levels with the exception of hyperparasitoids and earthworms for which knowledge was too limited. We specified a meaningful range for each parameter (Table 1) and divided these ranges linearly into 40 steps to represent small changes from low to high land use intensity. The ranges were based on expert knowledge provided by the authors and their colleagues at the Department of Terrestrial Ecology at the NIOO-KNAW. During the simulations, all gradient parameters were changed at the same time to acknowledge the simultaneous change of fertilization levels and searching efficiencies along real land use gradients.

### Simulated Removal Experiments

To investigate the direct and indirect influences of different trophic levels on plant performance along the land use intensity gradient, we designed removal scenarios. In each removal scenario, one trophic level was removed from the simulations (including earthworm removal). In total, this yielded seven removal scenarios including the standard scenario in which all levels were present. Removal can also be seen as a proxy for the application of a very effective pesticide. We performed simulated experiments with 1000 replicate runs per removal scenario and determined plant performance at the end of a growing season for each of the 40 steps along the land use intensity gradient. We measured plant performance as median shoot and root biomass of surviving plants at the end of a growing season and used plant mortality as an inverse proxy of plant performance and as a measure of instability. Plant mortality was calculated as the proportion of replicate runs in which the plant did not survive until the end of the growing season.

### Sensitivity Analysis of the Gradient Parameters

We conducted a sensitivity analysis to identify the gradient parameters with the greatest contribution to plant performance and to assess their relative contribution to the land use intensity gradient. Plant performance was measured as shoot biomass, root biomass and – inversely – as plant mortality. We ran 1000 replicate simulations each for every possible combination of plant performance measure and gradient parameter, varying the parameters one after the other within the ranges specified for the parameters. For each combination, we separately applied a linear regression model of the form performance ∼ gradient parameter value × removal scenario. We accounted for the removal scenario in the model formula to obtain sensitivity estimates that are independent of removal scenario. Sensitivity of the respective plant performance measure was calculated as standardized regression coefficient of the respective parameter value. Regression coefficients were standardized through division by the respective standard error.

### Statistical Analysis

To quantify the effects of trophic level removal on plant performance along the land use intensity gradient, we compared plant performance before and after removal with Pearson’s Chi-squared test statistic in the case of plant mortality and Mann-Whitney U-tests in the cases of root and shoot biomass. We used non-parametric tests and calculated the medians of root and shoot biomass, because non-normal errors and heterogeneous variance precluded calculating parametric statistics based on averages. We calculated the test statistics separately for each removal scenario (always comparing with the standard scenario) and for each of the 40 steps along the land use gradient. All statistical analyses were carried out with the software package R 2.5.1 (R Development Core Team 2007).

## Results

**Figure 1:**
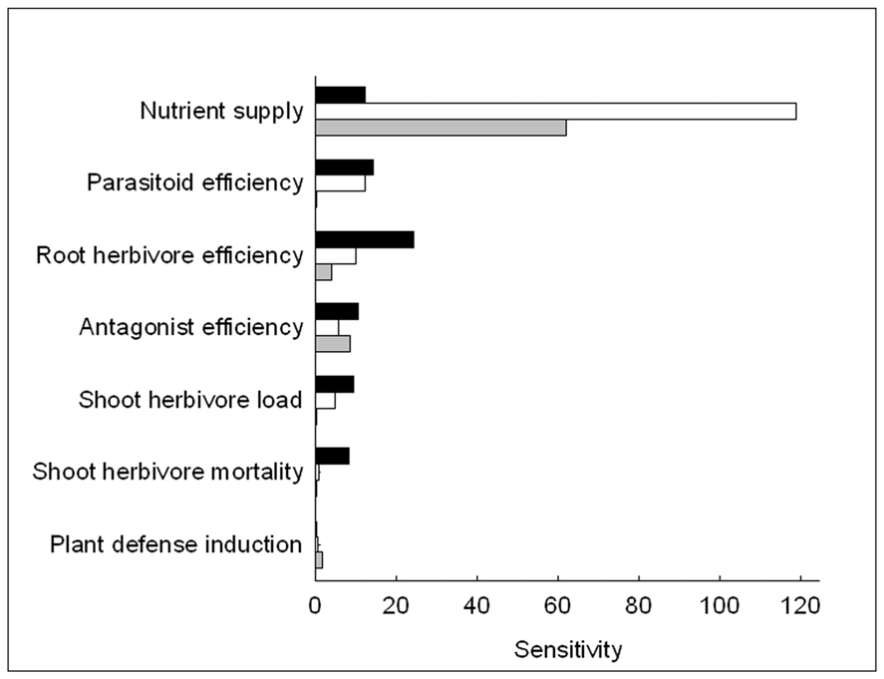
Sensitivity of model outputs to parameters of the land use intensity gradient. The model outputs were plant mortality (solid bars), average shoot biomass (empty bars), and average root biomass (grey bars). Sensitivity was derived from linear models of the form output ∼ gradient parameter * removal scenario for all possible combinations of outputs and gradient parameters. Sensitivity was calculated as the regression slope of the parameter divided by the corresponding standard error for standardization.

The sensitivity analysis showed that plant root and shoot biomass were by far most sensitive to the parameter nutrient supply (Fig. 1). Plant mortality was also sensitive to nutrient supply, but much more so to root herbivore efficiency and parasitoid efficiency. The sensitivity of all plant performance measures to all other tested parameters was relatively small.

**Figure 2:**
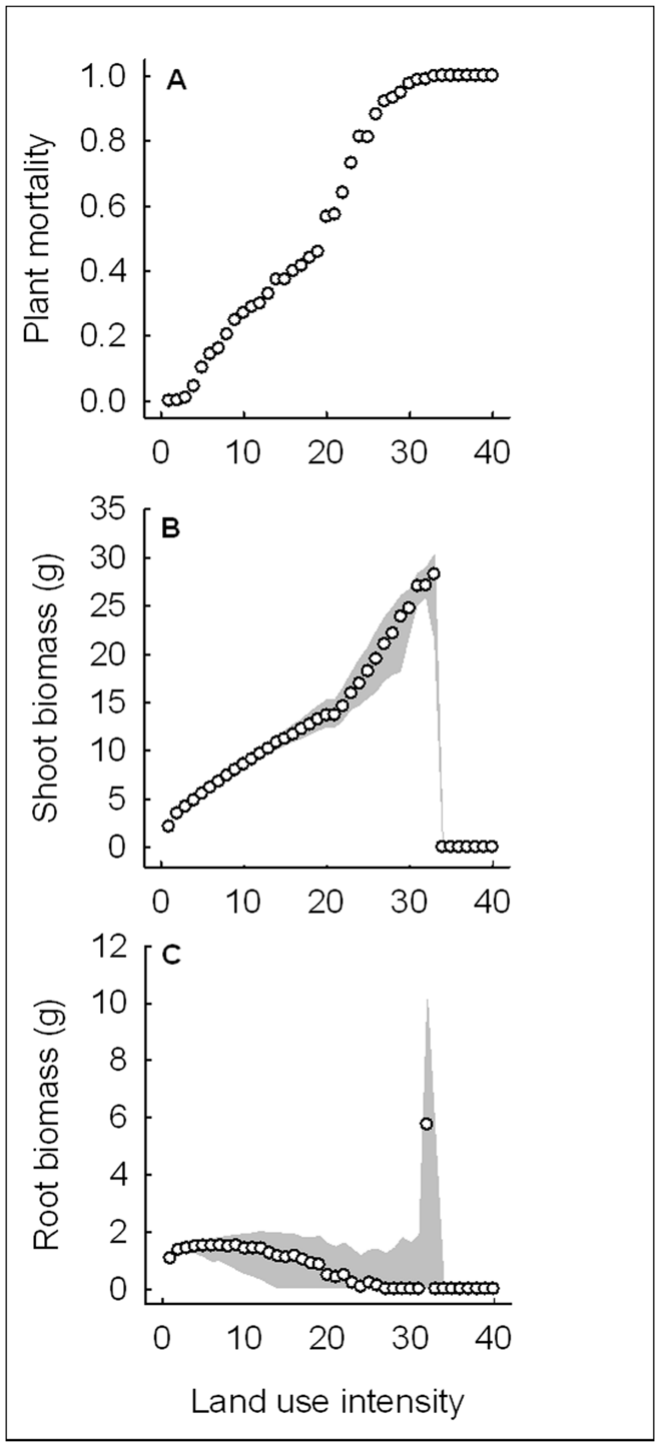
Plant performance with increasing land use intensity in the standard scenario. (a) Mortality and median (b) shoot and (c) root biomass of the model plant were based on 1000 simulation runs of the standard scenario. The land use intensity gradient was constructed by increasing nutrient supply, aboveground herbivore load, aboveground parasitoid efficiency, belowground herbivore efficiency, and belowground antagonist efficiency and decreasing plant defense induction and aboveground herbivore mortality in 40 steps within a meaningful range (Table 1). Mortality is the proportion of simulation runs in which the plant did not survive until the end of the growing season. Median shoot and root biomass are based on surviving plants only. Shaded areas indicate the interquartile range, i.e. contain 50% of the simulated data points. Note that the scales of the y-axes in (b) and (c) differ.

Plant mortality as a proxy of instability spanned the whole range from 0 to 1 (Fig. 2a). Plant mortality increased steeply and non-linearly with increasing land use intensity, approaching a plateau of 100% mortality in the quarter representing the most fertilized agroecosystems (Fig. 2a). Apart from this quarter, shoot biomass increased steeply with land use intensity and root biomass first remained stable at a low level and then decreased towards medium levels of land use intensity (Figs. 2b, c). Beyond the plant mortality level of 100%, root and shoot biomass reached zero, because the plant had died by the end of the simulations (see Fig. 2a). The transition to 100% plant mortality was sharp for shoot biomass but fuzzier for root biomass where some extreme values occurred (Fig. 2c). The interquartile ranges showed that the variability of shoot and root biomass was greatest at medium land use intensity (Figs. 2b, c).

**Figure 3:**
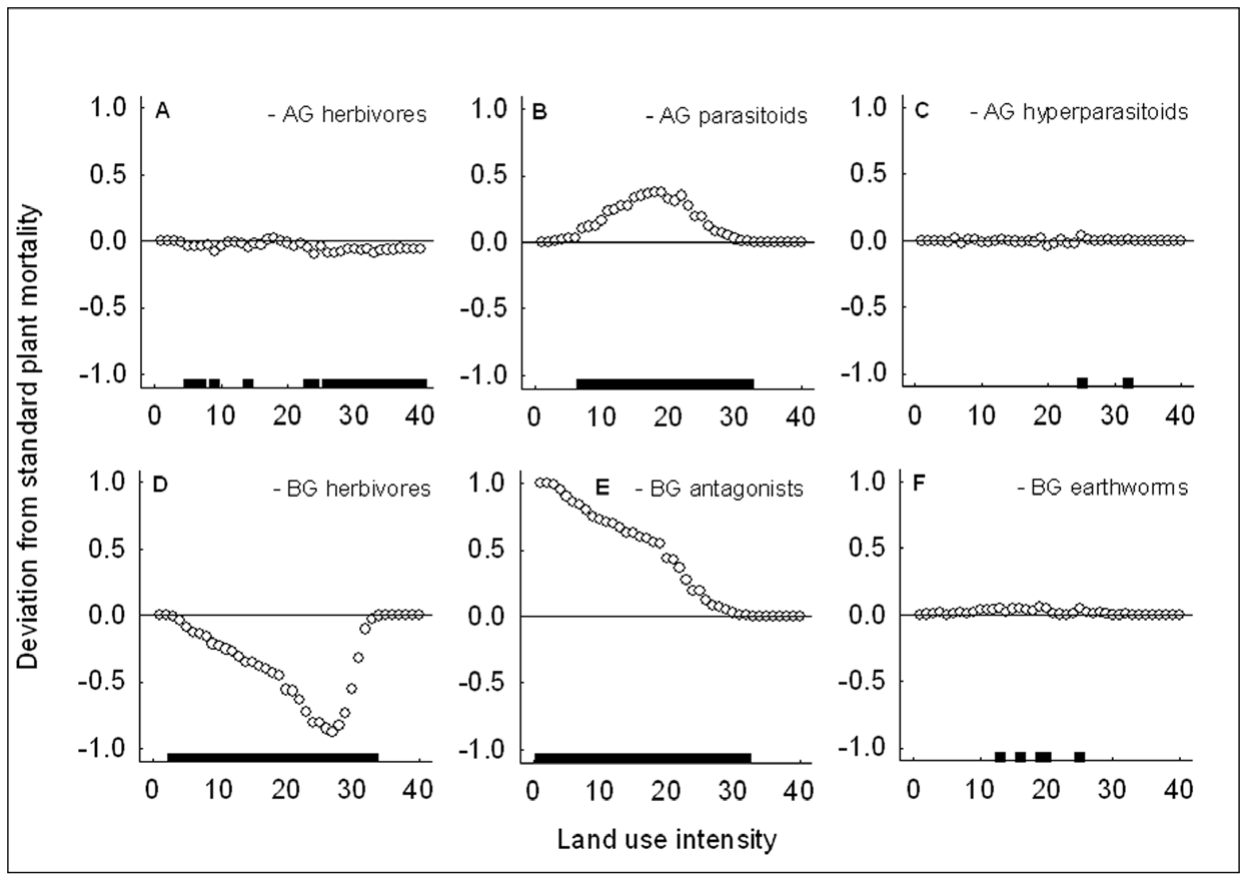
Plant mortality and multitrophic interactions with increasing land use intensity. Deviation from plant mortality in the standard scenario from low to high land use intensity after removal of aboveground (top row) and belowground (bottom row) trophic levels and earthworms, i.e. removal of aboveground (a) herbivores, (b) parasitoids, or (c) hyperparasitoids, or belowground (d) herbivores, (e) antagonists, or (f) earthworms. AG – aboveground, BG – belowground. The horizontal line at 0.0 represents the values of the standard scenario. Points indicate the difference in plant mortality between the respective removal scenario and standard scenario, and horizontal bars at the bottom of each graph indicate for each of the 40 points whether this difference is significant based on Pearson’s Chi-squared test statistic (*P*<0.05). Points above the 0.0-line show a positive effect of removal of the respective trophic level on plant mortality, points below the line a negative one. The horizontal bars show that belowground effects are more important under natural conditions while aboveground effects are stronger towards agroecosystems.

As expected, the removal of the second trophic level significantly reduced plant mortality at most land use intensities, i.e. herbivores diminished plant performance aboveground and belowground (see points below the zero-line in Figs. 3a, d). The removal of the third trophic level, the parasitoids and antagonists, significantly increased plant mortality aboveground and belowground (Figs. 3b, e), i.e. presence of the third trophic level increased plant performance. The removal of the fourth trophic level, the hyperparasitoids, and the earthworms, did not have a strong effect on plant mortality (Figs. 3c, f). Shoot herbivores were the only trophic level whose removal significantly reduced plant mortality below hundred per cent in highly fertilized agroecosystems (Figs. 3a). Aboveground herbivores had the strongest effect on plant mortality in these agricultural conditions, whereas belowground herbivores affected plant mortality more in natural to intermediate environments (compare position of horizontal bars along the gradient in Figs. 3a, d). Neither aboveground nor belowground third trophic level removal affected plant mortality at the 100% plant mortality plateau (Figs. 3b, e). However, the effect of aboveground parasitoids was more prominent at intermediate conditions while a significant effect of belowground antagonists also occurred under more natural conditions (Figs. 3b, e).

**Figure 4:**
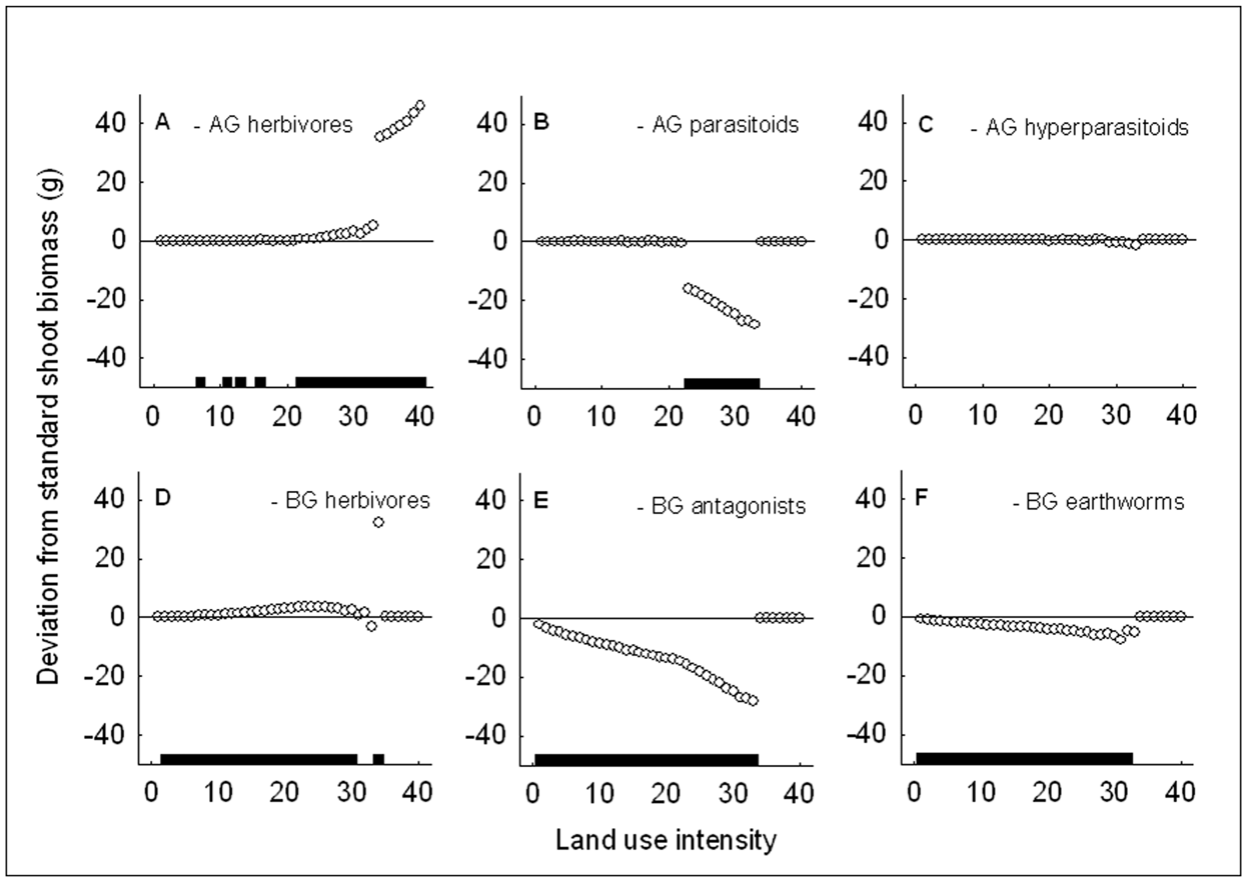
Shoot biomass and multitrophic interactions with increasing land use intensity. Deviation from median shoot biomass in the standard scenario from low to high land use intensity after removal of aboveground (top row) and belowground (bottom row) trophic levels and earthworms, i.e. removal of aboveground (a) herbivores, (b) parasitoids, or (c) hyperparasitoids, or belowground (d) herbivores, (e) antagonists, or (f) earthworms. AG – aboveground, BG – belowground. The horizontal line at 0.0 represents the values of the standard scenario. Points indicate the difference in shoot biomass between the respective removal scenario and standard scenario, and horizontal bars at the bottom of each graph indicate for each of the 40 points whether this difference is significant based on the Mann-Whitney *U*-test (*P*<0.05). Points above the 0.0-line show a positive effect of the removal of the respective trophic level on shoot biomass, points below the line a negative one. The horizontal bars show that belowground effects are more important under natural conditions while aboveground effects are stronger towards agroecosystems.

The effects of the removal of trophic levels on shoot biomass generally were in line with the effects on plant mortality, only their direction was opposite (Fig. 4). For instance, as expected, removal of herbivores had a significant positive effect on shoot biomass (Figs. 4a, d), whereas removal of the third trophic level had a significant negative effect (Figs. 4b, e). Earthworms did not have a strong effect on plant mortality, and their removal had a small negative effect on shoot biomass (Fig. 4f). The effect of shoot herbivores on shoot biomass was much greater than that of belowground herbivores (Figs. 4a, d). This revealed the potential biomass level that the plant could reach without aboveground herbivory in agroecosystems (Fig. 4a). The significantly positive effect of aboveground natural enemies of herbivores on shoot biomass was limited to intermediate land use intensities (Fig. 4b). For plant mortality, this effect was significant also at lower land use intensities (Fig. 3b).

**Figure 5:**
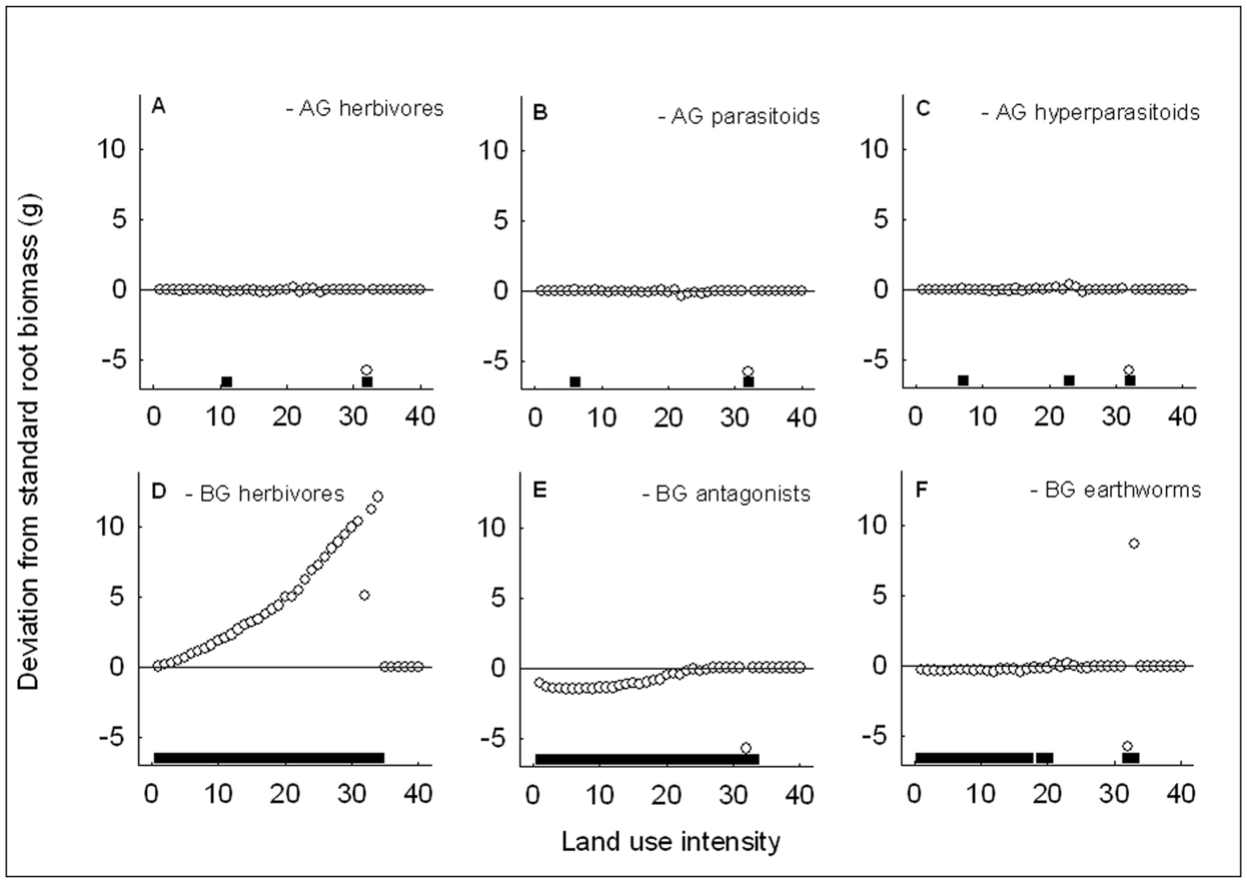
Root biomass and multitrophic interactions with increasing land use intensity. Deviation from median root biomass in the standard scenario from low to high land use intensity after removal of aboveground (top row) and belowground (bottom row) trophic levels and earthworms, i.e. removal of aboveground (a) herbivores, (b) parasitoids, or (c) hyperparasitoids, or belowground (d) herbivores, (e) antagonists, or (f) earthworms. AG – aboveground, BG – belowground. The horizontal line at 0.0 represents the values of the standard scenario. Points indicate the difference in root biomass between the respective removal scenario and standard scenario, and horizontal bars at the bottom of each graph indicate for each of the 40 points whether this difference is significant based on the Mann-Whitney *U*-test (*P*<0.05). Points above the 0.0-line show a positive effect of the removal of the respective trophic level on root biomass, points below the line a negative one. The horizontal bars show that belowground effects are dominating the effect on root biomass while aboveground effects are virtually absent.

In contrast to shoot biomass, root biomass was almost exclusively affected by belowground species (Fig. 5). Removal of belowground herbivores had a strong significantly positive effect on root biomass, and this positive effect increased from natural to intermediate conditions; of course this effect disappeared at 100% plant mortality (Fig. 5d). The effect of removing the belowground third trophic level was smaller with respect to root biomass than to shoot biomass (Figs. 4e and 5e). Removal of earthworms had a small negative effect on root biomass that was constant over the land use intensity gradient and disappeared at 100% plant mortality (Fig. 5f). Aboveground parasitoids did not affect root biomass (except for two single significant steps along the gradient, Fig. 5b).

## Discussion

Using an individual-based simulation model, we tested predictions derived from predator-prey theory [12] and specifically the paradox of enrichment [4] in a range of systems from natural communities to agroecosystems including multiple trophic interactions aboveground and belowground. Plant mortality increased steeply with increasing land use intensity, supporting our first prediction that systems should destabilize when fertilization and predator searching efficiencies are increased under changes in land use. We also found support for our second prediction, because belowground effects were more often significant under natural conditions and aboveground effects more often at high land use intensity. We found abrupt, non-linear responses of plant performance along the land use intensity gradient, rejecting the third prediction that changes in instability should be smooth if the underlying land use intensity gradient is smooth.

Consistent with our first prediction, plant mortality as a measure of instability increased towards highly fertilized agroecosystems in the presence of all aboveground and belowground trophic levels. Hence, the paradox of enrichment is still applicable in a system with multiple trophic levels in aboveground and belowground subsystems. Our finding implies that parasitoids and antagonists were not able to offset the negative effect of herbivores on plant performance. This was not a trivial outcome, because the gradient parameters included increased efficiencies of both herbivores and their natural enemies and the removal experiments showed negative effects of herbivores on plant performance and positive effects of natural enemies. It seems that shoot herbivores alone were responsible for the 100% plant mortality plateau (Fig. 3a compared to panels b–f), confirming their great potential for damaging highly fertilized crops. Our results on increasing herbivory with increasing fertilization and insect searching efficiencies are in line with previous findings. With respect to searching efficiencies, it has been shown that decreasing proportions of non-crop area in agricultural landscapes leads to increasing plant damage by rape pollen beetles [28] which may be due to greater insect searching efficiencies in crop-dominated areas. With respect to fertilization, phytophagous arthropod abundances have been found to increase with increasing land use intensity in tropical agroecosystems [29]. Hence, control of herbivores by their natural enemies seems to fail in highly fertilized or structurally poor environments.

Aboveground parasitoid removal gave more detailed insight into the efficiency of control of herbivores by their natural enemies along the land use intensity gradient: Aboveground biological control failed at high land use intensity and under natural conditions, but was successful in reducing plant mortality at intermediate land use intensities. With respect to shoot biomass loss, aboveground biological control was effective within an even smaller range located even further away from natural conditions (compare horizontal bars in Figs. 3b and 4b). This agrees well with the finding that parasitism of rape pollen beetles is at its maximum at 50% of non-crop area, corresponding to intermediate land use intensity [28] and supports the notion that strong aboveground top-down control is probably not a natural phenomenon (*sensu* [30]). In contrast, belowground natural enemies were effective at both low and intermediate land use intensities, indicating that belowground top-down control is probably largely independent of environmental context as long as land use intensity is not extremely high. This corresponds to greater control of herbivores exerted by soil organisms found in less managed systems [31]. This outcome may be accelerated if the attraction of natural enemies by volatiles emitted from damaged roots is considered [32]–[35] which may be integrated into future model versions. Our findings highlight that fertilization can to a certain extent be beneficial for successful biological control, especially aboveground, as long as it does not reach a level at which the herbivores and natural enemies are out of balance, allowing herbivores to dominate the system. Much of modern agriculture is characterized by such extremely high levels of fertilization, often necessitating the application of pesticides to correct for biological control failure if high yields are to be maintained.

Our results supported the second prediction that the strength of aboveground effects on system instability should increase towards agroecosystems. Effects of belowground trophic levels on plant mortality and biomass were more important at low land use intensities whereas aboveground trophic levels had a greater impact on plant performance at intermediate to high land use intensities. This was a general trend for all components of the community (compare the location of the horizontal bars in panels a versus d, b versus e and c versus f in Figs. 3, 4, and 5). Particularly the effect of shoot versus root herbivores is interesting and can be explained in terms of differences in population fluctuations. Shoot herbivores showed strong “boom-bust” population cycles with scramble competition [7], which can lead to rapid reduction of plant biomass in phases of booming population growth, but also rapid herbivore population crashes when resources are limited or parasitism gets too strong. In this case, the plant may survive while the herbivore population goes extinct. This is a typical case of too close coupling of predator and prey populations leading to instability and species extinction (*sensu* [8]). In contrast, root herbivores had much lower-amplitude population fluctuations. They contributed less to plant performance and mortality at high land use intensities. As an exception to this pattern, sedentary endoparasitic nematodes can produce great instability in agroecosystems by causing patches of decline in sugar beet and potato [36]. Nonetheless, the greater importance of belowground herbivores under more natural conditions is in line with findings of Blossey and Hunt-Joshi [37] and with evidence on the crucial role of root herbivores during early succession [38] which corresponds to low land use intensities.

Removal of the fourth trophic level, the hyperparasitoids, and of the earthworms did not have marked effects on plant performance. Only earthworm removal decreased shoot biomass with increasing land use intensity, which can be explained by model architecture: Presence of earthworms increases root and shoot biomass by a certain proportion of the current biomass and current biomass increased with increasing resource supply.

Our model analysis did not support our third prediction that the imposed smooth land use intensity gradient should result in smooth plant responses to trophic interactions. We observed abrupt non-linear effects of trophic level removal across all land use intensities and for all trophic levels. The most pronounced non-linearity was the rather suddenly reached 100% mortality plateau. Such enrichment-induced mortality and extinction have earlier been shown for plants in North America [39] and Great Britain [40] and for forest trees [41]. The few extreme values and the associated large variation in biomass that occurred at the transition to the 100% mortality plateau can be explained by model-inherent stochasticity that was inflated by the boom-bust dynamics of the herbivores. Just before reaching the 100% plant mortality plateau, these dynamics led to a broad range of possible biomass outcomes, also reflected in the few outliers at the same transition point in Figs. 4d and 5a–f. These observations are examples of alternative transient states [42] that are possible in dynamic systems under the same environmental conditions. Beyond the 100% plant mortality plateau, the great increase of shoot biomass in the absence of shoot herbivores revealed the immense potential growth that the plant could reach without aboveground herbivory at high land use intensity. This underlines how powerful aboveground herbivores with a boom-bust-population dynamical behavior can be as soon as fertilization exceeds a certain limit. It confirms the notion that the predictability and reliability of plant performance will decrease under enrichment. However, it also shows the great potential of aboveground biological control for crop yields, if natural enemies are supported to act as powerful control agents.

In conclusion, our model suggests that destabilization and extinction are more likely in fertilized agroecosystems than in natural communities, confirming the predictions of the paradox of enrichment also for communities with multiple aboveground and belowground trophic levels. Abrupt destabilization of the multitrophic system at high land use intensities implies that land use gradients as well as multiple trophic levels need to be considered to make reliable predictions on plant responses to land use change. These predictions will gain generality if more complex food webs with greater species diversities are considered in future modelling approaches. Based on our current predictions, it should be possible to tune land use in multitrophic agroecosystems such that the positive effect of natural enemies on plant performance is maximized while the risk of sudden transitions to high plant mortality is minimized.
